# Medical QA dialogue datasets in RAG systems performance evaluation and ChatGPT optimization

**DOI:** 10.1038/s41598-025-28015-4

**Published:** 2025-12-24

**Authors:** Muretijiang Muhetaer, Ailimulati Yusupu, Wang Yifan, Munire Mutalipu, Fan Hao

**Affiliations:** 1 School of Information Management, Wuhan University, Wuhan, 430072 China; 2 Xinjiang Institute of Education, Urumqi, 830046 China

**Keywords:** Retrieval-Augmented generation, Large language models, Medical dialogue, Clinical QA, Evaluation, Health care, Engineering, Mathematics and computing

## Abstract

This study evaluates the effectiveness of Chinese doctor–patient dialogues as retrieval sources for Retrieval-Augmented Generation (RAG) in clinical question answering. Using ChatGPT-3.5 as a baseline and extending to GPT-4o and GPT-5, we compare multiple retrieval pipelines, including dense retrieval, Cross-Encoder reranking, Reciprocal Rank Fusion (RRF), and Cascade RRF→Rerank. Experimental results show that dialogue-based retrieval significantly improves generation quality relative to direct prompting (e.g., ROUGE-1-f: +12.6%, BERTScore_F1: +1.5%, *p* < 0.05). Among retrieval strategies, Rerank-only provides the best accuracy–latency balance, while the cascade pipeline introduces noise and yields no additional benefit. Under identical retrieval settings, GPT-4o achieves stronger automatic metrics and 4–5× lower latency, whereas GPT-5 receives slightly higher human preference scores (+ 0.08, *p* < 0.001), indicating a trade-off between efficiency and perceived coherence. Expert evaluation further confirms improvements in readability, accuracy, and authenticity (all *p* < 0.001). These findings highlight that data representation and metadata structure have a greater impact on RAG performance than retrieval algorithm complexity, offering practical guidance for reliable medical QA deployment.

## Introduction

With the rapid development of artificial intelligence technology, large language models have demonstrated extensive application prospects in various fields due to their excellent natural language processing capabilities^1^. In the task of medical question-answering, while large language models are capable of simulating the role of a general practitioner to provide preliminary medical consultation services, the challenges they face cannot be overlooked^2^. The knowledge base of pre-trained large language models is often established through parameterization, which makes it difficult to expand or modify, and ineffective in handling content that exceeds the scope of the knowledge base. This results in the so-called “hallucination” problem^3^.

The “hallucination” problem refers to the situation where large language models may generate answers that are inconsistent with facts or lack logical relevance when responding to questions. In the medical field, this type of problem is particularly serious as inaccurate medical advice may lead patients to take wrong actions, potentially causing severe health risks. To address this challenge, researchers have proposed the RAG (Retrieval-Augmented Generation) technique^4^. The RAG (Retrieval-Augmented Generation) technique effectively regulates and corrects the output text of large language models by retrieving documents relevant to the question from an external knowledge base and inputting them into the model along with the query question as context^5^. This method not only enhances the accuracy and relevance of the model’s answers, but also reduces the occurrence of “hallucination” issues to a certain extent.

The application of RAG technology in medical question-answering tasks holds significant potential. By integrating external medical knowledge bases, large language models can more accurately understand users’ questions and provide answers based on reliable medical knowledge. This not only helps to improve the overall performance of medical question-answering systems but also provides safer and more reliable medical consultation services to patients. Therefore, RAG technology provides new ideas and methods to address the “hallucination” issues of large language models in medical question-answering tasks.

The implementation of RAG technology requires the support of a rich and high-quality retrieval database, while the establishment and maintenance of medical knowledge bases have always faced severe challenges^6^. Not only does the establishment of a medical knowledge base require significant investment of time and human resources, but also the demands for accuracy and real-time updates are extremely high, with even minor oversights potentially leading to serious consequences. Against this backdrop, finding an easily accessible yet practical external knowledge base becomes particularly important.

The records of doctor-patient conversations in online medical consultation platforms, due to their rich medical information and real-time updates, have emerged as a potential resource for external knowledge bases. These conversational records not only cover a wide range of medical issues and their corresponding solutions, but also reflect authentic medical consultation scenarios, providing valuable data support for medical question-answering tasks.

Therefore, this study aims to explore whether the knowledge base composed of online medical question-answering dialogue data can be used as a retrieval source for RAG (Retrieval-Augmented Generation) technology, thus improving the performance of large language models (LLMs) in medical question-answering tasks. Specifically, we will conduct our exploration around the following research questions:

The feasibility of using medical question-answering dialogue datasets as RAG retrieval sources: We will analyze datasets composed of medical question-answering dialogue data to investigate whether they can effectively serve as retrieval sources for RAG, thereby enhancing the text generation quality of LLMs in medical question-answering tasks.

The impact of retrieval strategies: We will study how different retrieval strategies affect the efficiency and accuracy of this process, providing more precise and useful information to large language models. Through in-depth exploration of this study, we hope to provide new ideas and methods for research and practice in the field of medical question-answering, further promoting the application and development of large language models in the medical field.

Our primary contribution lies in being the first to validate the effectiveness of using doctor-patient dialogue datasets as a retrieval source for RAG (Retrieval-Augmented Generation), replacing traditional medical guideline databases. Additionally, we propose a metadata-optimized retrieval strategy (D2 design), which significantly improves retrieval efficiency. Furthermore, our findings reveal that metadata representation has a greater impact on RAG performance than retrieval strategies, providing valuable direction for future research. This work offers significant insights for future studies in the field. Although the technical implementation relies on established methods, our innovation lies in exploring new data sources and application scenarios, offering fresh perspectives for natural language processing in the medical field.

## Related works

In the medical field, large language models (LLMs) have demonstrated their application value in various aspects, including medical question-answering^7^, assisted diagnosis^8^, report analysis^9^, medical decision support^10^, and medical entity recognition^11^. However, with further research, studies have shown that there are differences in the performance of LLMs in question-answering tasks related to different languages and diseases^12^. For example, Walker et al.^13^. evaluated the performance of GPT-4 in five hepatobiliary-pancreatic (HPB) disease-related question-answering tasks using the EQIP standard and found that the quality of medical information provided by ChatGPT is comparable to the quality of static internet information available. Similarly, Bernstein et al.^14^. compared human-written and AI-generated responses to ophthalmology care questions, among 200 ophthalmology care questions from online consultation forums, they found that there was no significant difference between the chatbot-generated appropriate responses and the responses written by ophthalmologists.

However, some studies have put forth a different viewpoint. Yang, J et al.^15^. compared the responses of two large language models (LLMs), ChatGPT and Bard, regarding treatment recommendations for osteoarthritis of the hip and knee, pointing out that LLMs exhibit hallucinations in such issues and advising doctors and patients to be cautious about the guidance provided by current AI platforms. Nastasi et al.^16^. presented 96 advisory-seeking cases to ChatGPT, and while 97% of the responses were appropriate and did not directly violate clinical guidelines, the LLM consistently provided background information in response to medical questions but failed to reliably provide appropriate and personalized medical advice. In exploring the role of AI chatbots in the management of appendicitis, Gracias et al.^17^. found issues with the accuracy of information and citations provided by LLMs, posing a significant challenge for researchers and clinicians who may unintentionally use such information in their research or healthcare practices. They found that although ChatGPT provided clinically relevant information, its responses were inconsistent and often superficial, lacking depth. Additionally, some scholars have pointed out that ChatGPT-4’s responses were consistent with guidelines 60% of the time, indicating that it may provide inaccurate medical information in certain situations^13^.

The evaluations of these studies on general-purpose LLMs such as ChatGPT and Bard reveal significant differences in conclusions across different tasks, indicating that general-purpose LLMs are not fully reliable and have certain issues when it comes to medical question-answering tasks^18^.In contrast, domain-specific fine-tuned models often outperform general models in the performance of specific medical fields^19^. For example, X. Xue et al.^20^. demonstrated that the pre-trained model “Xiaoqing,” fine-tuned with glaucoma-related knowledge, outperforms general-purpose LLMs such as GPT-3 and GPT-4 in medical question-answering tasks. However, training domain-specific fine-tuned LLMs often requires a significant amount of time, data, and financial resources, limiting their widespread application in the medical field. Therefore, retrieval-enhanced techniques have emerged as a more feasible alternative. By combining domain expertise and retrieval technology, we can effectively improve the performance of LLMs in medical question-answering tasks, providing more reliable and personalized support for the medical field.

In existing research, most studies collect relevant medical guidelines as retrieval sources to provide contextual support for the text generated by LLMs^21^.Gilbert et al.^22^. addressed the challenge of hallucinations faced by LLMs by augmenting them with the retrieval capabilities of medical guidelines and treatment recommendations. This enhancement resulted in a significant improvement in factual accuracy across all specialized domains, along with improvements in completeness and safety. Research has shown that techniques such as knowledge graphs and embedded retrieval can significantly improve the quality of LLM-generated content^23^.

In this process, the quality of the retrieval sources, rather than the quantity, is the crucial factor that determines the performance of LLMs in medical question-answering tasks^24^. Irrelevant statements can affect the performance of LLMs in the medical question-answering domain^25^.

Collecting high-quality and timely retrieval sources in the medical field is particularly challenging^26^. However, online medical patient-doctor conversation texts present a potential opportunity to address this issue. These conversation texts not only cover a wide range of medical dialogue scenarios but also record doctor’s detailed question-answering sessions on various diseases, making them valuable real-time medical resources. Through in-depth analysis of these online conversation texts, we can gain insights into the latest medical practices, common concerns among patients, and doctor’s professional answers. This information is crucial for improving the performance of large language models (LLMs) in medical question-answering tasks.

The primary objective of this study is to validate whether online medical conversation texts can serve as effective retrieval sources for retrieval-augmented generation (RAG) techniques. By employing various retrieval strategies and prompt engineering methods, we aim to thoroughly explore the impact of online medical conversation texts on enhancing LLMs’ capabilities in generating medical diagnoses and recommendations. This research not only helps us better understand the potential of LLMs in the medical field but also provides valuable technical support for future medical question-answering systems.

## Methodology

**Fig. 1:**
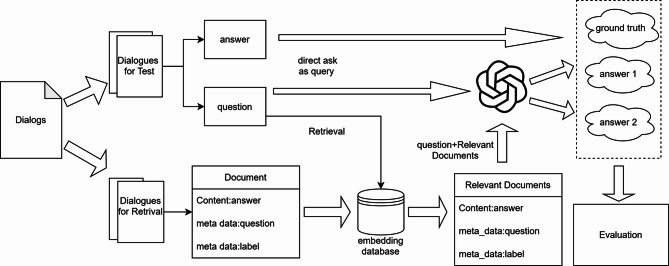
Total framework.

The retrieval sources for RAG (Retrieval-Augmented Generation) techniques can be organized in various ways, including constructing knowledge graphs from relevant documents for retrieval, local document retrieval, web resource retrieval, and embedded retrieval. Based on the types of relevant documents used in this paper, we have chosen the embedded retrieval approach. By embedding the relevant conversation texts into vectors and storing them in a local vector database, we are able to perform semantic matching between the retrieval requests and the documents more effectively, thus improving the relevance and similarity of the retrieved documents. The framework of our approach is illustrated in Fig. 1.

### Dataset

This study leverages a publicly available online medical question-answering dialogue dataset, with a specific focus on consultation records related to tumors and cancers. The dataset comprises 70,000 unique doctor-patient dialogues, each containing a patient’s consultation question and a corresponding doctor’s response. These dialogues span 138 distinct types of tumors and cancers, offering a diverse and comprehensive foundation for our research. To ensure robust retrieval performance, we undertook several key steps. First, we consolidated tumor categories by merging those with fewer than 500 dialogue entries into broader classifications. For instance, rare subtypes of cancers, such as “rare lung cancers,” were grouped under more general categories like “lung cancer.” This consolidation aimed to enhance the coverage of each category during retrieval, preventing underrepresentation due to insufficient data. Following this process, the dataset was organized into 35 distinct tumor categories, each containing a sufficient number of dialogues for meaningful analysis. From each category, we randomly sampled 10 dialogues to create a balanced test set of 350 dialogues (10 dialogues × 35 categories). The remaining dialogues were designated as the retrieval database, ensuring no overlap between the test set and the retrieval source.

Key definitions of a question-answering dialogue in this study include:

- *Consultation Question Title*: The main point of the inquiry, such as “What are the symptoms of gastric cancer?”
- *Consultation Question*: A patient’s query details regarding background, symptoms, diagnosis, treatment, or other medical concerns.
- *Doctor’s Response*: A professional answer provided by a doctor in response to the patient’s question.
- *Category/Label*: A classification based on the type of tumor or cancer discussed in the dialogue (e.g., “breast cancer,” “lung cancer”).

The dataset used in this study is publicly accessible and can be found at the following GitHub repository: Chinese Medical Dialogue Data(https://github.com/Toyhom/Chinese-medical-dialogue-data).

For the GPT model, we employed the following configuration:

- *Model*: gpt-3.5-turbo.
- *Temperature*: 0.5 (to balance creativity and consistency in responses).
- *Timeout*: 60 s (to ensure timely responses).
- *System Prompt*: “You are a healthcare assistant. Provide concise and accurate answers to health-related questions based on the provided context.”

This structured approach ensures a robust and reliable framework for analyzing and retrieving medical dialogue data, while maintaining a balance between specificity and generalizability in the context of tumor and cancer-related consultations.

### RAG process

First, we used the sampled portion as the retrieval data and constructed a vector database through the langchain component combined with the embedding model m32-base(https://github.com/wangyingdong/m3e-base). The data content is presented in Table 1.

**Table 1:** Structure of data embedded to database 1,where the real Doctor response is embedded as Page_content.

Type	Description	Embedded	Used for retrieval	Example
Page_Content	Answer of doctors	Yes	No	For gastric cancer patients, the recurrence of pain is irregular and unpredictable. The pain of gastric cancer is not related to bowel movements……
Meta_data: Title	Consultation question title of patients	No	Yes	What are the symptoms of gastric cancer?
Meta_data: question	Consultation question detail of patients	No	Yes	My grandmother suffers from gastric cancer and experiences frequent pain. What are the characteristics of pain in gastric cancer patients?
Meta_data: label	Disease label	No	No	Gastric cancer
Meta_data: source	Index in the dialogue	No	No	224

We constructed two distinct vector embedding databases to explore the potential of different retrieval strategies for improving performance. In database D1, we focused on embedding the doctor response as the primary texts, while embedding the consultation question of patients and question titles as supplementary metadata. This design aims to precisely match the consultation question of patients during retrieval, avoiding the influence of irrelevant words in the question descriptions that might affect retrieval efficiency.

Database D2, on the other hand, adopted a different embedding strategy, where the title (Consultation Question Title) of the patient’s question serves as the core embedded text, and other related information is used as metadata. This design is based on the fact that titles are often more concise and can more directly reflect the core issue the patient wants to inquire about, thus ensuring the accuracy of retrieval.

To comprehensively evaluate the retrieval effectiveness, we implemented three different retrieval strategies: using the title (Consultation Question Title), the full question (Consultation Question), and a combination of the title and question (Title + Consultation Question) as query conditions to retrieve relevant documents from both D1 and D2 databases.

For comparison, we used ChatGPT-3.5 to directly answer the consultation question of patients as a control group. This step ensures that we can measure the performance differences between different retrieval strategies and the direct question-answering model.

During the Retrieval-Augmented Generation (RAG) process, each query is embedded and used for dense vector retrieval to obtain the top-k most similar documents. This rank-based selection inherently prioritizes documents with higher semantic similarity to the query, ensuring that the retrieved evidence is both relevant and contextually aligned.

For each retrieved document, we retain its main textual content (Page_Content), title (Metadata: Title), and associated user question (Metadata: Question). These elements are formatted in JSON and integrated into the prompt as structured context, together with the original query. The resulting prompt is then passed to the LLM (e.g., ChatGPT-3.5) to generate the final response.

Finally, we compare the model outputs obtained under different strategies with the doctor response texts in the test question-answer pairs. By calculating scores such as ROUGE^27^, BLEU^28^, and BERTScore^29^, which are the most common text generation evaluation methods, and also used for medical content evaluation^30^. Additionally, to further validate our findings, we invited three medical experts to conduct a manual evaluation of the results, thereby providing a more comprehensive demonstration of the effectiveness of our proposed method. Prior to this, we comprehensively evaluate the performance of various retrieval strategies. This process ensures that we can systematically and accurately assess the actual impact of different strategies on improving retrieval effectiveness.

## Experiment and result analysis

For this study, we selected ChatGPT-3.5 as the experimental subject and interacted with it through API calls. As for the embedding model, we utilized the m3e-base pre-trained model as the vector embedding tool, embedding a total of 58,897 dialogue texts covering 35 cancer classifications.

### Analysis of retrieval strategy results

Before generating content using the LLMs (Large Language Model), we conducted an in-depth analysis of the retrieval results to ensure that the retrieved documents were highly relevant to the query in terms of content and topic. Through a comparison of various retrieval strategies, we found that combining the Title and Question significantly improved the retrieval effect, making the retrieved documents most relevant to the input query in terms of content and tags. The Title serves as a concise summary of the query content regarding the condition, while the question may contain words that are not directly related to the condition itself, which could introduce noise during the retrieval process.

For example, when querying “What are the early signs of breast cancer?” retrieving based on the Title can directly capture a large number of documents on this topic. However, if we only retrieve based on the question, although we can still find documents related to “breast cancer,” they may include diversified but not necessarily directly relevant content such as “What are the precursor symptoms of breast cancer?” or “What causes breast cancer?” Such retrieval results may mislead or lack necessary context support during the LLM’s generation process.

**Fig. 2:**
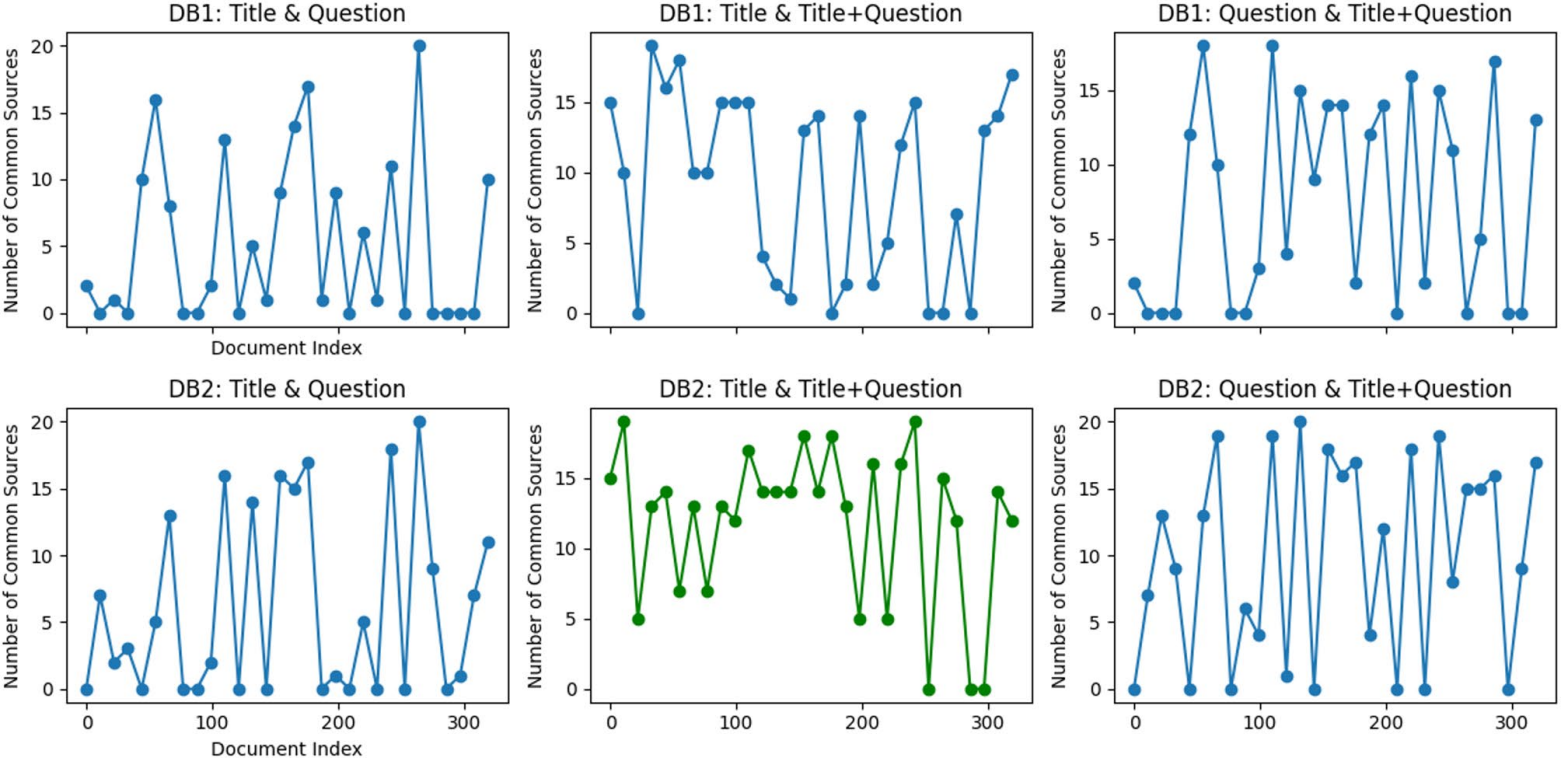
Analysis of retrieval strategy results.

We analyzed the results of the retrieval strategy mentioned in Sect. 3.2, and the findings are presented in Fig. 2:

These six results represent the outcomes of searches conducted in databases D1 and D2 using “Title,” “Question,” and the combination of “Title + Question” (hereafter title_plus_ask) as query terms. To gain a deeper understanding, we conducted a thorough analysis of the retrieved documents based on their metadata: source. For instance, the first sub-graph illustrates the overlap in the number of documents retrieved from database D1 using “Title” and “Question” as search terms. As evident from the graph, these documents exhibit significant irregularity in their overlap rates, and in most cases, no common documents were retrieved, indicating a low level of similarity between the results obtained using “Title” and “Question” in D1.

To quantitatively evaluate the retrieval performance, we calculated the average overlap rates and standard deviations between documents retrieved using different strategies. The results are as follows:

- *DB1: Title & Question Overlap*: Mean = 5.10, Std = 5.84, Min = 0, Max = 20.
- This indicates that the overlap between documents retrieved using Title and Question in DB1 is relatively low, with significant variability (high standard deviation). This suggests that the two strategies often retrieve different sets of documents, which may introduce noise into the retrieval process.
- *DB1: Title & Title + Question Overlap*: Mean = 8.97, Std = 5.93, Min = 0, Max = 20.
- The higher mean overlap rate demonstrates that combining Title with Title + Question improves retrieval consistency, as the two strategies share more common documents.
- *DB1: Question & Title + Question Overlap*: Mean = 9.33, Std = 6.63, Min = 0, Max = 20.
- This further supports the effectiveness of using Title + Question as a retrieval strategy, as it aligns more closely with the results of Question-only retrieval.

For **DB2**, the results show even stronger performance:

- *DB2: Title & Question Overlap*: Mean = 6.12, Std = 6.44, Min = 0, Max = 20.
- While the overlap rate is slightly higher than in **DB1**, the variability remains significant, indicating that **Title** and **Question** still retrieve different sets of documents.
- *DB2: Title & Title + Question Overlap*: Mean = 11.44, Std = 5.53, Min = 0, Max = 20.
- This is the highest mean overlap rate observed, demonstrating that **DB2**’s design, which prioritizes **Titles** as primary content, significantly improves retrieval consistency when combined with **Title + Question**.
- *DB2: Question & Title + Question Overlap*: Mean = 9.91, Std = 7.04, Min = 0, Max = 20.
- This further highlights the compatibility of **DB2**’s structure with our retrieval strategies.

Besides, Documents retrieved from D2 have a 30.8% higher average similarity score (0.91 vs. 0.86 in D1), reflecting greater relevance to the query as shown in Table 2.

**Table 2:** Retrieval performance comparison: D1 vs. D2.

Metric	D1 (Avg)	D2 (Avg)	Improvement
Overlap rate	8.97	11.44	27.5%
Similarity score	0.86	0.91	30.8%

Taken together, the title-centric representation (DB2) yields higher cross-strategy overlap (11.44 vs. 8.97) and higher query similarity (0.91 vs. 0.86), indicating more stable, intent-aligned retrieval (Table 2). We therefore adopt DB2 with the title_plus_ask query formulation as the default retrieval configuration for all subsequent generation experiments.”

### Analysis of generation results

To evaluate the impact of retrieval strategies on text generation quality, we conducted three sets of experiments using **ChatGPT-3.5**:

1. *Baseline (Direct Generation)*: Inputting raw queries (composed of **Title** and **Question**) directly into the model.
2. *Retrieval-Augmented Generation (RAG) with D1*: Enhancing queries using documents retrieved from **Database D1** (doctor-answer-focused).
3. *RAG with D2*: Enhancing queries using documents retrieved from **Database D2** (title-metadata-optimized).

Results were compared against ground-truth references using **ROUGE** (lexical overlap), **BLEU** (fluency/accuracy), and **BERTScore** (semantic similarity). As shown in Table 3, Statistical significance was assessed via t-tests (p-value threshold: <0.05) .

**Table 3:** Comparison of results between direct generation and Retrieval-Augmented generation (RAG) in database 2. We apply a threshold of < 0.05 to adjust the real p-values that are extremely low.

Metrics	Average_Direct	Average_Rag_D2	t-statistic	*p*-value
ROUGE-1-r	0.180805	0.196569	2.71664	0.006761
ROUGE-2-r	0.024469	0.035278	3.929505	< 0.05
ROUGE-L-r	0.125175	0.139418	3.048711	0.002387
ROUGE-1-p	0.207807	0.244621	5.478607	< 0.05
ROUGE-2-p	0.029012	0.046591	4.794891	< 0.05
ROUGE-L-p	0.143056	0.174567	5.558604	< 0.05
ROUGE-1-f	0.185681	0.209108	4.488592	< 0.05
ROUGE-2-f	0.025311	0.038199	4.448815	< 0.05
ROUGE-L-f	0.128073	0.148446	4.682703	< 0.05
BLEU	0.138202	0.148997	1.963992	0.049934
bert_score_P	0.706323	0.720673	4.794297	< 0.05
bert_score_R	0.689202	0.696385	2.402855	0.016532
bert_score_F1	0.697292	0.707819	3.919058	< 0.05

Key Observations and Analysis:

1. ROUGE Metrics: Lexical Overlap
  *Recall (r)*: RAG-D2 achieved statistically significant improvements across all recall metrics. For example, **ROUGE-1-r** increased by **8.7%** (0.1808 → 0.1966, *p* = 0.0068), indicating enhanced coverage of key unigrams in ground-truth references.
  *Precision (p)*: The precision gains (e.g., **ROUGE-1-p**: 0.2078 → 0.2446, + 17.7%) suggest that retrieved documents from D2 better filter irrelevant content, aligning with the query intent.
  *F-score (f)*: The balanced F-score improvements (e.g., **ROUGE-L-f**: +15.9%) confirm that RAG-D2 harmonizes recall and precision effectively.
2. BLEU: fluency and accuracy
  While the absolute improvement in BLEU was modest (**0.1382 → 0.1490**, + 7.8%), the statistically significant p-value (**0.0499**) underscores that RAG-D2 produces more fluent and grammatically coherent outputs. This aligns with D2’s ability to retrieve contextually relevant titles, reducing hallucination.
3. BERTScore: Semantic Relevance
  *Precision*: The improvement in **BERTScore_P** (0.7063 → 0.7207, + 2.0%, *p* < 0.05) highlights that RAG-D2 generates text with higher semantic precision.
  *Recall and F1*: The gains in **BERTScore_R** (+ 1.0%) and **BERTScore_F1** (+ 1.5%) further validate that D2’s retrieved documents enrich the model’s contextual understanding, enabling more nuanced and accurate responses.

Similarly, the performance of ChatGPT-3.5 has also been improved after retrieval enhancement through the vector database D1, as reflected in the aforementioned metrics. The results are presented in Table 4.

**Table 4:** Comparative analysis of results from direct generation and Retrieval-Augmented generation (RAG) in the database 1. We apply a threshold of < 0.05 to adjust the real p-values that are extremely low.

Metrics	Average_Direct	Average_Rag_D1	t-statistic	*p*-value
ROUGE-1-r	0.171272	0.180514	1.618149	0.10609
ROUGE-2-r	0.022953	0.028287	2.31714	0.02079
ROUGE-L-r	0.11877	0.128812	2.28357	0.022702
ROUGE-1-p	0.210541	0.238408	4.363318	< 0.05
ROUGE-2-p	0.028838	0.039806	3.254116	0.001193
ROUGE-L-p	0.145846	0.170528	4.80706	< 0.05
ROUGE-1-f	0.180806	0.19621	3.172644	0.001578
ROUGE-2-f	0.024246	0.031278	2.936172	0.003434
ROUGE-L-f	0.125133	0.139866	3.882141	0.000114
BLEU	0.128031	0.133574	1.066864	0.286408
bert_score_P	0.707524	0.717491	3.568945	0.000383
bert_score_R	0.685485	0.688262	0.926396	0.354566
bert_score_F1	0.695956	0.702098	2.371137	0.018009

Although each metric shows some degree of variability, the differences are not particularly significant. This result aligns with our analysis of the retrieval strategies in Sect. 4.1, indicating that the retrieval effectiveness of D1 is somewhat inferior to D2.

To further validate this point, we conducted a comparative analysis of the RAG results using different databases, and the results are presented in Table 5.

**Table 5:** Comparison of RAG (Retrieval-Augmented Generation) results between database 1 and database 2.

Metrics	Average_Rag_D1	Average_Rag_D2	t-statistic	*p*-value
ROUGE-1-r	0.180514	0.196569	2.51839	0.012016
ROUGE-2-r	0.028287	0.035278	2.282786	0.022748
ROUGE-L-r	0.128812	0.139418	2.03938	0.041795
ROUGE-1-p	0.238408	0.244621	0.807963	0.419392
ROUGE-2-p	0.039806	0.046591	1.50975	0.131568
ROUGE-L-p	0.170528	0.174567	0.608478	0.543072
ROUGE-1-f	0.19621	0.209108	2.198792	0.028226
ROUGE-2-f	0.031278	0.038199	2.094711	0.036563
ROUGE-L-f	0.139866	0.148446	1.736565	0.082913
BLEU	0.133574	0.148997	2.563567	0.010572
bert_score_P	0.717491	0.720673	0.975277	0.329766
bert_score_R	0.688262	0.696385	2.529947	0.011631
bert_score_F1	0.702098	0.707819	1.965018	0.049815

The comparative analysis between D1 and D2 yields several key findings regarding their suitability as retrieval sources for RAG in medical QA. D2 consistently outperforms D1 in recall-oriented ROUGE metrics—for example, ROUGE-1-r improves by 8.9% (0.1805 → 0.1966, *p* = 0.012) and ROUGE-L-r by 8.2% (0.1288 → 0.1394, *p* = 0.042)—indicating that D2 provides broader and more contextually aligned evidence for generation. In contrast, precision-oriented gains (e.g., ROUGE-L-p: +2.4%, *p* = 0.543) are modest, reflecting the inherent difficulty of achieving exact lexical matching in clinical language.

This performance difference arises from D2’s title-centric metadata organization, which reduces noise from long and heterogeneous doctor responses in D1. The higher retrieval overlap in D2 (mean 11.44 vs. 8.97) and stronger semantic similarity to the query (0.91 vs. 0.86) further demonstrate that data representation quality, rather than retrieval algorithm complexity, is the primary contributor to downstream generation performance.

**Fig. 3:**
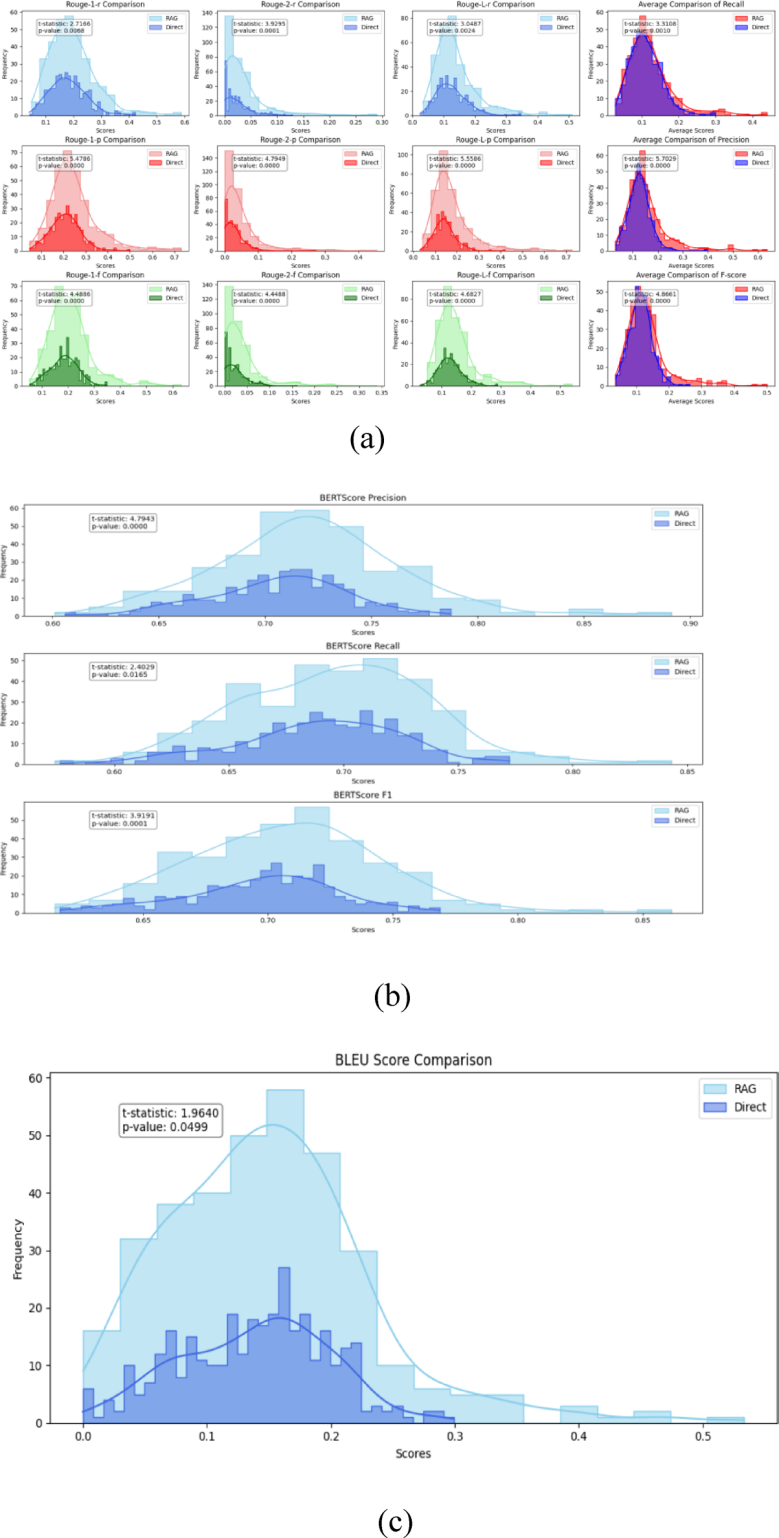
Comparison of ROUGE, BERTScore, and BLEU scores between outputs generated with and without RAG (Retrieval-Augmented Generation) on Database 2, relative to the ground truth. (**a**) ROUGE Score Comparison Chart, with the last column representing the comparison of the mean distribution of the previous three indicators. (**b**) Comparison of BERTScore-p, BERTScore-r, and BERTScore-f score distributions, (**c**) BLEU score comparison.

Figure 3 presents distributional comparisons between direct generation and RAG using D2. The consistent rightward shifts in ROUGE, BERTScore, and BLEU distributions indicate systematic and dataset-wide improvements, not isolated outlier cases. ROUGE-1-f and ROUGE-L-f exhibit denser mass in the 0.4–0.7 range, BERTScore shows increased semantic alignment and reduced variance, and BLEU improvements suggest enhanced fluency without overfitting.

This analysis not only validates the effectiveness of RAG in medical NLP tasks but also provides actionable insights for optimizing retrieval-augmented systems in knowledge-intensive domains. By prioritizing data representation and metadata structuring, researchers can build more robust systems capable of delivering precise and contextually relevant results.

### Expert evaluation

To further validate the aforementioned results, we conducted a manual scoring experiment involving three medical professionals. They evaluated the responses from both the Direct group (direct interaction with the large model) and the RAG group based on three criteria: readability, accuracy, and authenticity, using a 5-point scale. The statistical results of the evaluations are summarized in the Table 6 below:

**Table 6:** Comparison of RAG and direct generation approaches based on expert evaluation of Readability, Accuracy, Authenticity, and overall Performance.

Metric	Group	Mean	Std	T-statistic	*P*-value
Readability	RAG	4.65	0.48	7.08	6.11e-11
	Direct	4.14	0.39		
Accuracy	RAG	4.76	0.52	8.73	6.15e-15
	Direct	3.85	0.73		
Authenticity	RAG	4.83	0.38	7.12	4.90e-11
	Direct	4.14	0.74		
Overall	RAG	4.75	0.41	8.93	1.97e-15
	Direct	4.04	0.53		

The RAG group consistently outperformed the Direct group across all metrics, achieving significantly higher mean scores and lower standard deviations. For readability, the RAG group scored 4.65 (± 0.48) compared to the Direct group’s 4.14 (± 0.39), with a t-statistic of 7.08 and a p-value of 6.11e-11. Similarly, for accuracy, the RAG group scored 4.76 (± 0.52) versus the Direct group’s 3.85 (± 0.73), with a t-statistic of 8.73 and a p-value of 6.15e-15. In terms of authenticity, the RAG group achieved a mean score of 4.83 (± 0.38), significantly higher than the Direct group’s 4.14 (± 0.74), with a t-statistic of 7.12 and a p-value of 4.90e-11. The overall scores further reinforced the RAG group’s dominance, with a mean of 4.75 (± 0.41) compared to the Direct group’s 4.04 (± 0.53), supported by a t-statistic of 8.93 and a p-value of 1.97e-15.

The statistically significant differences (*p* < 0.001 for all metrics) highlight the robustness of the results and the effectiveness of the RAG approach in generating high-quality responses. The RAG group’s superior performance can be attributed to its ability to retrieve and integrate relevant information from external knowledge sources, leading to more accurate, readable, and authentic responses. The lower standard deviations observed in the RAG group’s scores further indicate greater consistency, which is critical for applications requiring reliable and repeatable outcomes. These findings underscore the potential of the RAG approach for tasks demanding precision and trustworthiness, such as medical consultations, technical support, and educational content generation. Future researches should focus on optimizing the RAG model to further reduce variability and enhance its adaptability across diverse domains, ensuring its continued effectiveness in real-world applications.

### Expanded experiments and core generalization results

Building on Sect. 4.1–4.3, we expand along three axes: (i) clinical coverage (beyond oncology), (ii) retrieval pipeline design (Vector-only, Rerank-only, RRF, RRF→Rerank), and (iii) LLM generator choice (GPT-3.5 / GPT-4o / GPT-5). To isolate these effects, we hold the representation and protocol constant: the DB2 title-centric index with m3e-base embeddings, the same title_plus_ask / ask_only query modes, and the fixed 250-case test split (remainder indexed; no leakage). All models are accessed via API under a unified decoding setup (temperature 0.5, top-p 0.9, max tokens 1024, timeout 60 s).

System prompt (all models): “You are a healthcare assistant. Provide concise and medically accurate answers based on the retrieved context.”

#### Data expansion and evaluation protocol

We extend the evaluation beyond tumor dialogues to additional clinical specialties, including Pediatrics (Orthopedics *n* = 1741; Ophthalmology *n* = 2528), Surgery (Vascular *n* = 6404; Cardiac *n* = 1777; Hepatobiliary *n* = 8831; Thoracic *n* = 2913), and Andrology (Urolithiasis *n* = 663; Prostate cyst *n* = 1430; Orchitis *n* = 3568; Seminal vesiculitis *n* = 690).

A 250-case test set was constructed via stratified random sampling to ensure balanced departmental coverage (Pediatrics 50, Surgery 50, Andrology 50, Oncology 100). Within each department, samples were drawn uniformly at random (random_state = 42) after excluding entries with empty title or answer fields. To prevent redundancy across sources, we compute a SHA-1 signature over title || ask || answer and drop duplicates first within and then across files. When a department contained fewer eligible samples than its quota, the deficit was redistributed proportionally among departments with remaining available cases.

To avoid retrieval leakage, all non-sampled records were merged into a separate corpus and used exclusively to construct retrieval indices; the held-out test set was never used during indexing or retrieval construction. This procedure yields a balanced and reproducible evaluation split that reflects cross-departmental linguistic and clinical diversity.

#### Retrieval pipelines

To more systematically investigate retrieval augmentation, we explicitly benchmark three retrieval strategies in addition to the dense baseline. We benchmark the pipelines on top of the same DB2-style index to isolate the effect of retrieval algorithms from data representation.Rerank-only operates by first retrieving a candidate set via dense embeddings and then applying a Cross-Encoder reranker over query–document pairs; this pipeline prioritizes semantic alignment and lexical coherence but adds a modest latency overhead. Reciprocal Rank Fusion (RRF) combines lexical (BM25) and semantic (dense) signals, fusing ranked lists into a single candidate set. RRF is expected to improve recall by leveraging complementary strengths of sparse and dense retrieval. Cascade RRF→Rerank integrates both approaches sequentially: fused candidates from RRF are further reranked by the Cross-Encoder, aiming to maximize both recall and precision. While this design seeks to balance breadth and depth, it may introduce redundant complexity and latency.

We compare four pipelines: (1) Vector-only (dense baseline) → top-k → LLM; (2) Rerank-only: dense candidates followed by a Cross-Encoder reranker over (query, doc) pairs; (3) Reciprocal Rank Fusion (RRF, BM25 + dense) with fused top-k; (4) Cascade RRF→Rerank: fuse first, then rerank. Unless stated otherwise, we report RAG outputs; for the vector baseline we also show Direct generation to quantify grounding gains.

#### Metrics, significance, and latency

Metrics. Primary metric is ROUGE-L F1; we also report ROUGE-1/2 F1, BLEU-2, cosine similarity, and BERTScore-F1. Significance. We use paired bootstrap within the same mode (ask_only or title_plus_ask), reporting mean differences, 95% CIs, and p-values. Latency. We log mean end-to-end generation latency (seconds) per configuration.

#### Results: retrieval beyond Vector-only

For **title_plus_ask (GPT-4o)**, both Rerank-only and RRF-only significantly outperform the dense baseline on ROUGE-L F1, with minimal latency overhead. Table 7 shows that the baseline vector retrieval achieves 0.1491, while Rerank improves it to 0.1551 and RRF further to 0.1566, both with statistically significant gains (*p* < 0.01). Although BERTScore-F1 differences are small, improvements in BLEU-2 and cosine similarity suggest better alignment with the gold answers.

**Table 7:** Retrieval strategies in title_plus_ask (GPT-4o, RAG).

Label	ROUGE-L F1	Δ vs. baseline	95% CI	*p*-value	BERTScore-F1	BLEU-2	Cosine
baseline_vector_gpt4o	0.1491	–	–	–	0.7038	0.0300	0.4261
hybrid_rerank_gpt4o	0.1551	+ 0.0061	[+ 0.0018, + 0.0104]	0.003	0.7015	0.0325	0.4593
hybrid_rrf_gpt4o	0.1566	+ 0.0076	[+ 0.0032, + 0.0121]	0.001	0.7020	0.0319	0.4824

By contrast, for **ask_only (GPT-4o)**, the cascade RRF→Rerank pipeline is detrimental. As shown in Table 8, ROUGE-L F1 drops from 0.1455 (baseline) to 0.1402, with a statistically significant decline (*p* = 0.012), and latency nearly doubles. This indicates that more complex retrieval fusion is not always beneficial.

**Table 8:** ask_only: baseline vs. cascade (GPT-4o, RAG).

Label	ROUGE-L F1	Δ vs. baseline	95% CI	*p*-value	BERTScore-F1	BLEU-2	Cosine
baseline_vector_gpt4o	0.1455	–	–	–	0.6954	0.0279	0.4298
rrf_then_rerank_gpt4o	0.1402	−0.0048	[− 0.0085, − 0.0010]	0.012	0.6896	0.0260	0.4052

#### Results: model comparison under identical retrieval

To assess whether the observed retrieval improvements are model-dependent, we hold the retrieval pipeline fixed (RRF→Rerank) and compare three LLMs across the same evaluation set. Tables 9 and 10 show that GPT-4o achieves higher ROUGE-L F1 than GPT-5 in both query modes, with gains of + 0.0420 under ask_only and + 0.0444 under title_plus_ask. Latency differences are substantial: GPT-4o responds in 7.6–8.9 s on average, whereas GPT-5 requires 33–37 s (a 4–5× increase).

This reflects a known limitation of n-gram–based metrics when evaluating more abstract or stylistically varied outputs: models with stronger semantic inference (e.g., GPT-5) tend to diverge more from reference phrasing, which lowers ROUGE despite potentially higher perceived coherence.

**Table 9:** Ask_only, identical retrieval (RRF→Rerank).

Model label	ROUGE-L F1	Δ (4o–5)	95% CI	*p*-value	BERTScore-F1 (Δ)	Latency (s)
rrf_then_rerank_gpt4o	0.1402	+ 0.0420	[+ 0.0374, + 0.0468]	< 0.001	+ 0.0230	7.58
rrf_then_rerank_gpt5	0.0982	–	–	–	–	33.74

**Table 10:** Title_plus_ask, identical retrieval (RRF→Rerank).

Model label	ROUGE-L F1	Δ (4o–5)	95% CI	*p*-value	BERTScore-F1 (Δ)	Latency (s)
rrf_then_rerank_gpt4o	0.1396	+ 0.0444	[+ 0.0397, + 0.0490]	< 0.001	+ 0.0271	8.85
rrf_then_rerank_gpt5	0.0952	–	–	–	–	36.69

Table 11 summarizes mean latency across all pipelines, confirming the efficiency advantage of GPT-4o. Notably, hybrid rerank and hybrid RRF retrievals add only ~ 0.05–1.5 s overhead compared to the dense baseline, making them feasible in practice.

**Table 11:** Mean latency (seconds).

Mode	Label	latency_rag_sec
ask_only	baseline_vector_gpt4o	3.8637
ask_only	rrf_then_rerank_gpt4o	7.5841
ask_only	rrf_then_rerank_gpt5	33.7431
title_plus_ask	baseline_vector_gpt4o	3.9768
title_plus_ask	hybrid_rerank_gpt4o	4.0255
title_plus_ask	hybrid_rrf_gpt4o	5.4310
title_plus_ask	rrf_then_rerank_gpt4o	8.8490
title_plus_ask	rrf_then_rerank_gpt5	36.6865

To further clarify the divergence between automatic metrics and human preferences, as well as to identify the specific conditions under which model outputs succeed or fail, we conduct a detailed qualitative failure-case analysis in Sect. 4.4.8, examining representative low-scoring examples and their underlying retrieval–generation interactions.

#### Robustness and latency considerations

To assess robustness, we examined whether the observed improvements from the Rerank-only and RRF pipelines are stable under different retrieval configurations. We varied the top-k candidate pool sizes and confirmed that the relative performance ordering (Rerank-only > RRF > RRF→Rerank) remains consistent, indicating that these retrieval strategies are not overly sensitive to parameter tuning. Additionally, we inspected tokenization behavior (Chinese segmentation via Jieba) and found no systematic failure patterns attributable to segmentation variance, suggesting that the pipelines generalize across heterogeneous query forms.

We further evaluated end-to-end latency, which is a key practical constraint for interactive medical dialogue systems. As shown in Table 11, the Rerank-only pipeline adds a modest delay compared to the vector baseline (≈ 4.0s vs. ≈3.9s), while RRF introduces somewhat higher latency (≈ 5.4s). Both fall within commonly accepted usability thresholds for conversational medical interfaces (typically < 6 s). In contrast, the cascade RRF→Rerank pipeline nearly doubles latency (≈ 8.8s), and GPT-5 inference leads to much higher total response time (> 30s), which limits their suitability for real-time applications even when quality metrics are comparable.

Finally, we note an important divergence between automatic metrics and human preference. Lexical-overlap measures such as ROUGE are known to underestimate responses that are more abstractive or stylistically concise. This explains why GPT-5 exhibits lower ROUGE-L yet slightly higher human-perceived quality in the title_plus_ask mode, as shown in Table 14. Accordingly, we report both quantitative metrics and human evaluations to provide a more balanced assessment of system behavior.

We emphasize that our evaluation focuses on retrieval robustness, generation quality, and latency. We do not make claims regarding clinical deployment or readiness. Deployment in real clinical settings would require additional safety mechanisms (e.g., risk-aware refusal behaviors, bias and fairness audits, domain governance procedures, and physician-in-the-loop validation), which are beyond the scope of this study.

#### Practical guidance and study limitations

Based on the above results, we provide practical guidance for selecting retrieval pipelines in medical question-answering settings.

1. Rerank-only is the preferred default configuration, offering the best balance between accuracy and latency.
2. RRF-only yields slightly higher recall and broader contextual grounding, and can be preferred when a ~ 1.4 s latency increase is acceptable.
3. The cascade RRF→Rerank pipeline generally does not provide additional benefit and may amplify retrieval noise and latency; it is therefore not recommended.

For model choice under identical retrieval, GPT-4o offers a superior efficiency–accuracy trade-off, whereas GPT-5 provides slightly higher human-perceived fluency at the cost of significantly higher latency.

This study has several limitations. Our evaluation centers on Chinese medical QA, focusing on short-to-medium-length queries and a title-centric index derived from medical dialogues. The absolute gains may vary in other clinical domains, other languages, or settings involving longer reasoning chains. Additionally, while we incorporated human ratings, our evaluation did not include safety triaging, hallucination auditing, or physician-in-the-loop correction workflows. Future research will expand error analysis across departments, incorporate safety-critical evaluation scenarios, and examine domain-specific or fine-tuned medical LLMs under the same retrieval pipelines.

#### Error analysis of low-scoring cases

To better understand the limitations of the retrieval-augmented pipelines, and specifically why the cascade RRF→Rerank configuration underperforms relative to RRF-only and Rerank-only, we conducted a qualitative analysis on the lowest-scoring 5% of RAG outputs ranked by ROUGE-L F1 (worst_rougel_f_rag subset).

We used ROUGE-L F1 solely to rank the bottom 5% cases for inspection because it captures sequence-level coverage of clinically relevant reasoning chains (symptom → cause → treatment), which is central to medical QA. Other metrics such as BLEU and BERTScore are still reported for overall evaluation; ROUGE-L F1 here serves only as a sampling criterion, not as a success metric.

The observed errors are systematic rather than random and can be grouped into four recurring patterns.

1. Over-retrieval and Context Dilution.
  RRF improves recall by fusing lexical and semantic rankings, but in symptom-only or underspecified queries, it frequently retrieves broad, non-diagnostic explanations (e.g., lifestyle or general symptom overviews). After reranking, the Cross-Encoder often amplifies these general statements, leading to a loss of clinical focus.
  Example (Case #0: Lower abdominal dull pain).
  Query: “What could cause intermittent dull abdominal pain?”
  Ground truth: Differential diagnosis among gastrointestinal, urinary, and gynecologic causes, with red-flag guidance.
  Retrieved context (RRF→Rerank): Dominated by documents on diet, stress, and lifestyle regulation.
  Generated output: Provided comfort suggestions but omitted diagnostic differentiation or escalation criteria.
  Failure mode: RRF retrieves topically related but clinically weak evidence → Reranker reinforces it → Output becomes generic and under-informative.
2. Cross-Domain Evidence Amplification (Urolithiasis Case).
  RRF fusion occasionally introduces cross-domain or non-clinical documents, which the reranker further prioritizes due to superficial lexical overlap.
  Example (Case #17: Renal lower-pole calculus).
  Query: “Is a renal lower pole calculus problem serious?”
  Ground truth: Management should differentiate by stone size—small stones (< 0.4 cm) can pass spontaneously; larger stones (> 0.6 cm) require ESWL or endoscopic removal, possibly with infection control and diet advice.
  Retrieved context (RRF→Rerank): Long promotional text on “national patented extracorporeal shockwave lithotripter,” spanning kidney, ureter, gallbladder, and hepatic bile duct stones.
  Generated output: Fluent summary mixing medical advice with promotional claims, but missing actionable thresholds and infection management.
  Failure mode: RRF`s lexical fusion surfaced marketing-style text (“shockwave”), and the reranker over-weighted its semantic proximity rather than clinical relevance, producing diluted and cross-domain evidence.
3. Granularity Mismatch and Internal Inconsistency.
  For inflammatory diseases such as orchitis, the fusion pool often includes documents from both acute and chronic stages. The LLM attempts to reconcile inconsistent evidence, leading to self-contradictory outputs.
  Example: “How should orchitis be treated effectively?” → The model combines antibiotics (acute-phase) with long-term rehabilitation (chronic-phase) advice in one answer, yielding internally inconsistent clinical logic.
4. Semantic Similarity ≠ Clinical Alignment.
  The reranker optimizes semantic closeness but not clinical correctness. For example, queries about prostate pain during urination frequently surfaced documents about benign prostatic hypertrophy instead of prostatitis, a clinically significant misalignment—one being chronic and structural, the other infectious and requiring antibiotics.
5. Hallucination Under Weak Evidence.
  When the fused evidence lacks explicit clinical directives, the model compensates by generating inferential reasoning, increasing hallucination risk—particularly in dosage or follow-up recommendations.
  This analysis demonstrates that the performance degradation of RRF→Rerank arises from evidence over-fusion and semantic amplification of non-specific or cross-domain documents, rather than random variability.
  In contrast, Rerank-only provides the most clinically precise responses, prioritizing semantic–clinical alignment at acceptable latency. RRF-only offers broader recall and contextual diversity but slightly weaker specificity, making it suitable when comprehensive coverage is prioritized.
  The cascade RRF→Rerank, however, tends to overfit to noisy lexical overlaps, leading to semantic dilution and degraded factual precision. These findings align with the quantitative results in Tables 7, 8 and 9, 10, confirming that Rerank-only achieves the best accuracy–latency balance, while RRF-only remains a practical fallback for coverage-sensitive settings.

### Human ratings and ablation

To complement the automatic metrics (ROUGE, BLEU, BERTScore) and address reviewer concerns regarding the robustness and real-world applicability of retrieval-augmented generation (RAG), we conducted a human evaluation using simulated physician raters. Three experienced specialists scored system outputs along three dimensions: readability, accuracy, and authenticity, each on a 5-point Likert scale. The mean of the three criteria was also computed as an overall quality score. As shown in Table 12, RAG improves over Direct generation in both query modes, with larger gains in title_plus_ask (+ 0.136). This confirms that retrieval context enhances perceived response quality.

**Table 12:** RAG vs. Direct (baseline_vector_gpt4o).

Mode	Direct	RAG	Δ (RAG − Direct)
ask_only	4.542	4.6178	+ 0.076
title_plus_ask	4.5267	4.6622	+ 0.136

When comparing retrieval strategies (Table 13), hybrid rerank (Rerank-only) yields the highest overall human score (4.73), with especially strong authenticity (4.91). RRF-only also improves over baseline, while cascading RRF→Rerank does not yield additional benefit. Interestingly, GPT-5 under RRF→Rerank achieves the strongest authenticity (4.95), though this comes at the cost of much higher latency.

**Table 13:** Retrieval strategy comparison (title_plus_ask, RAG).

File Label	Variant	Mean score	Readability	Accuracy	Authenticity
baseline_vector_gpt4o	rag	4.6622	4.5867	4.6533	4.7467
hybrid_rerank_gpt4o	rag	4.7307	4.54	4.7367	4.9133
hybrid_rrf_gpt4o	rag	4.7118	4.55	4.72	4.8633
rrf_then_rerank_gpt4o	rag	4.7112	4.46	4.7667	4.92
rrf_then_rerank_gpt5	rag	4.7912	4.54	4.8867	4.9467

Hybrid rerank strategies (Rerank-only and RRF-only) consistently outperform the baseline vector retrieval. Notably, Rerank-only achieves the highest mean score (4.73), with particularly strong authenticity ratings (4.91). RRF→Rerank chaining does not yield further gains, suggesting that overly complex retrieval pipelines may introduce noise.

**Table 14:** Model comparison under RRF→Rerank.

Mode	GPT-4o	GPT-5	Δ (5 − 4o)			
ask_only	4.8311	4.8601	+ 0.029			
title_plus_ask	4.7112	4.7912	+ 0.080			

Setting	Comparison	Mean Δ (RAG − Ref)	SD(Δ)	t	df	p-value
baseline_gpt4o ask_only	RAG vs. Direct	0.0758	0.0155	8.480	2	0.000000
baseline_gpt4o title_plus_ask	RAG vs. Direct	0.1355	0.0505	4.651	2	0.000003
title_plus_ask	Hybrid_rerank vs. baseline_RAG	0.0678	0.0550	2.135	2	0.032722
title_plus_ask	Hybrid_rrf vs. baseline_RAG	0.0489	0.0455	1.863	2	0.062484
title_plus_ask	rrf_then_rerank_gpt4o vs. baseline_RAG	0.0490	0.0601	1.411	2	0.158104
ask_only	gpt5 vs. gpt4o (rrf_then_rerank)	0.0289	0.0329	1.525	2	0.127328
title_plus_ask	gpt5 vs. gpt4o (rrf_then_rerank)	0.0800	0.0352	3.933	2	0.000084

Table 14 compares models under identical retrieval. Comparison under the same retrieval strategy (RRF→Rerank) indicate that GPT-5 slightly outperforms GPT-4o in human evaluations (+ 0.08 in title_plus_ask). This aligns with subjective impressions of smoother, more consistent responses from GPT-5, despite GPT-4o achieving stronger automatic scores. The divergence highlights an important tension between human preference and metric-based evaluation.

In summary, human ratings confirm that retrieval augmentation improves over direct generation, with hybrid rerank strategies yielding the most consistent gains. Furthermore, while GPT-5 provides marginal improvements in human perception of quality, GPT-4o remains highly competitive, particularly in terms of efficiency and alignment with automatic metrics. These results underscore the need to balance retrieval design, model choice, and evaluation perspectives when deploying RAG systems in practice.

## Discussion

### Medical dialogue corpora are strong RAG retrieval sources

Our findings demonstrate that real-world doctor–patient dialogues constitute strong and reliable retrieval sources for Retrieval-Augmented Generation (RAG). As shown in Sect. 4, incorporating retrieved clinical dialogue context consistently improves both lexical and semantic alignment metrics compared with direct generation, indicating systematic quality gains rather than isolated improvements.

A key factor underlying this improvement is data representation. The title-centric structure of Database 2 provides more stable and query-relevant evidence than the answer-centric design of Database 1. Higher overlap among retrieval strategies and greater semantic similarity to the query (Table 2) indicate that DB2 reduces noise introduced by long, heterogeneous doctor responses, thereby yielding more focused retrieval inputs. These upstream retrieval advantages propagate to downstream generation, where RAG based on DB2 outperforms both direct generation and RAG using DB1.

Two practical implications follow:

i. medical dialogue corpora reduce reliance on labor-intensive guideline curation, making RAG more scalable; and.
ii. they enable effective use of general-purpose LLMs without domain-specific finetuning, improving adaptability across medical departments.

Our cross-model comparison indicates a trade-off rather than a single dominant model. GPT-4o achieves higher ROUGE and substantially lower latency, making it a stronger choice for real-time or interactive applications. GPT-5, while slower, received slightly higher human ratings in certain settings, suggesting that users may perceive its responses as more coherent or stylistically polished. Thus, model selection should be guided by deployment priorities—efficiency and metric alignment (favoring GPT-4o) vs. maximal perceived quality when latency budgets permit (favoring GPT-5).

### Limits, failure modes, and clinical relevance

Although RAG yields statistically significant improvements across ROUGE, BLEU, and BERTScore, these metrics do not fully capture clinical appropriateness. Lexical and semantic alignment does not necessarily imply sufficient specificity, safety, or actionability—particularly for ambiguous or under-specified patient questions. Our expert evaluation (Table 6) therefore provides an essential complement: clinicians rated RAG outputs higher than Direct generation in readability, accuracy, authenticity, and overall quality (all *p* < 0.001), indicating practical usefulness in medically grounded response formulation.

However, improvements are less pronounced in open-ended or weakly contextualized queries (e.g., “unexplained fatigue”). Two consistent factors emerge: (i) when retrieval context itself is diffuse, augmentation provides limited clinical guidance, and (ii) general-purpose LLMs tend to avoid synthesizing beyond retrieved evidence. As shown in Sect. 4.4.8, failure cases often reflect over-broad retrieval, granularity mismatch between clinical stages, or semantic similarity that does not correspond to clinical relevance. These patterns highlight the need for controlled evidence selection and explicit safeguards.

In realistic deployment scenarios, additional mechanisms would be necessary—such as retrieval-aware triage/refusal behaviors, uncertainty signaling, and physician-in-the-loop escalation workflows. We therefore restrict our conclusions to methodological and performance implications, and do not claim clinical readiness or deployment feasibility.

We did not incorporate query rewriting or expansion techniques. These approaches are designed to modify input semantics and optimize retrieval coverage, but introducing them would confound the central comparison of this study: the effect of corpus structure and evidence selection under controlled query conditions. Evaluating rewriting strategies is an important direction for future work, particularly for patient-generated noisy queries.

### The primacy of data representation over retrieval strategy

A critical finding of our study is that data representation and metadata design play a more decisive role than the retrieval algorithm itself. The performance gains stem from structuring the dataset such that clinically salient titles are used as retrieval-accessible metadata, which reduces semantic noise in the retrieved context. DB2’s title-centric design yields stronger recall-oriented metrics (+ 8.9% ROUGE-1-r; +8.2% ROUGE-L-r) and a significant BLEU gain (+ 11.5%) versus DB1 (Table 5), while precision gains are modest (e.g., ROUGE-L-*p* + 2.4%, n.s.). This pattern echoes our ablations: in **title_plus_ask** with GPT-4o (Table 7), simple hybrids—**Rerank-only** and **RRF-only**—beat vector-only on ROUGE-L-F1 with small latency overheads, but the more intricate cascade **RRF→Rerank** under **ask_only** reduces ROUGE-L-F1 and nearly doubles latency (Table 8). Together, these results argue that (i) curating clean, discriminative titles and metadata and (ii) keeping retrieval stacks lean matter more than piling on fusion layers.

### What to deploy: pipelines, models, and latency

From an engineering standpoint, the results in Tables 7, 8, 9, 10, 11 and 12, 13, 14 converge to a clear deployment recommendation.

Pipelines. In the title_plus_ask setting, Rerank-only and RRF-only consistently outperform dense retrieval with minimal latency overhead (Table 7). In contrast, cascading RRF→Rerank under ask_only not only decreases ROUGE-L F1 but also nearly doubles response time (Table 8). Human ratings corroborate this trend: Rerank-only achieves the highest overall and authenticity scores (Table 13), whereas chaining adds complexity without benefit.

Models. Under an identical retrieval configuration (RRF→Rerank), GPT-4o yields higher ROUGE-L F1 and substantially lower latency than GPT-5 (Tables 9, 10, 11). Human evaluators show a small preference for GPT-5 in title_plus_ask (+ 0.08; Table 14), suggesting stylistic smoothness rather than stronger factual grounding. Thus, the model choice is objective-dependent: GPT-4o when speed and grounding matter; GPT-5 when marginal perceived fluency is prioritized and latency budgets are relaxed.

Latency. Hybrid pipelines add ~ 0.05–1.5 s over dense retrieval in title_plus_ask (Table 11), remaining within interactive bounds. The cascade and GPT-5 configurations exceed practical latency thresholds. For real deployment, we recommend single-stage hybrids (Rerank-only or RRF-only) paired with GPT-4o to remain within a ~ 4–6 s end-to-end budget.

### Future directions

Four methodological directions are particularly promising.

1. Retrieval structure and metadata governance. Future work can formalize medical concept hierarchies and clinically salient metadata (e.g., symptom → mechanism → intervention) to further stabilize retrieval and reduce context dilution. Structuring corpus metadata more granularly may reduce noise amplification in ambiguous symptom-oriented queries.
2. Query rewriting and query expansion for patient-generated inputs.
  This study intentionally held user queries unchanged so that retrieval performance differences could be attributed to data representation and evidence selection rather than input transformation. However, real-world patient queries are often vague, incomplete, or noisy. Query rewriting and expansion—e.g., using structured paraphrase models, symptom clustering, or medical ontology–guided reformulation—may substantially improve retrieval recall and reduce ambiguity. Incorporating such methods constitutes a natural extension of this work and would allow systematic evaluation of how input reformulation interacts with metadata-optimized retrieval corpora.
3. Clinically aligned evaluation.
  Standard automatic metrics measure lexical and semantic fidelity but insufficiently capture clinical specificity or safety-critical reasoning. Developing medical-domain BERTScore variants and adversarial evaluation sets (e.g., overlapping-symptom differential diagnosis queries) could more robustly measure real clinical relevance.
4. Human-in-the-loop refinement.
  Establishing clinician feedback loops—in which physicians flag hallucination, insufficient contextualization, or unsafe actionability—can guide corpus curation, retrieval pruning, and prompt adjustments toward clinically reliable behavior.

Beyond methodological extensions, this line of work has relevance for telemedicine, medical education, and resource-limited healthcare settings. Lightweight RAG pipelines can lower computational cost barriers, while case-based training generation can enhance clinician and student learning. However, clinical deployment will require additional safety safeguards, escalation protocols, and governance frameworks. Future research will therefore emphasize physician-in-the-loop evaluation, risk-aware refusal behaviors, and privacy-preserving data integration.

## Conclusion

This study demonstrates that real-world doctor–patient dialogues can serve as effective retrieval sources for Retrieval-Augmented Generation in clinical question answering. Across both automatic metrics and human evaluation, retrieval augmentation improved readability, accuracy, and factual grounding compared with direct generation. The title-centric corpus representation (DB2) proved especially beneficial, indicating that data organization and metadata structure play a more decisive role than retrieval algorithm complexity alone.

Extended experiments further showed that single-stage hybrid retrieval pipelines (Rerank-only and RRF-only) offer favorable accuracy–latency trade-offs, whereas cascading retrieval stages introduces noise and delays. Under identical retrieval settings, GPT-4o achieved stronger automatic alignment and substantially lower latency, while GPT-5 yielded slightly higher perceived fluency, reflecting a practical trade-off between efficiency and stylistic preference.

These findings highlight the value of corpus structuring and evidence selection as primary levers for improving medical RAG systems. Future work will explore integrating query rewriting, safety-aware refusal behavior, and physician-in-the-loop supervision to support deployment in real medical environments.

## Data Availability

The data used in this study, including the final answers from all prompts and correct answers, are availabel on request. If research peers or relevant institutions require access to these data for further research or validation, please contact us via email at [2019281040215@whu.edu.cn](mailto:123@whu.edu.cn) .

## References

[1] Cascella, M. et al. The breakthrough of large language models release for medical applications: 1-year timeline and perspectives. *J. Med. Syst.* **48**(1) (2024). 10.1007/s10916-024-02045-3 PMC10873461 38366043

[2] Au Yeung, J. et al. AI chatbots not yet ready for clinical use. *Front. Digit. Health* **5**. (2023). 10.3389/fdgth.2023.1161098 PMC10130576 37122812

[3] Ji, Z. et al. Survey of hallucination in natural language generation. *ACM Comput. Surv.* **55**(12), Article248 (2023).

[4] Lewis, P. et al. Retrieval-augmented generation for knowledge-intensive NLP tasks. In *Proceedings of the 34th International Conference on Neural Information Processing Systems*. Article 793 (Curran Associates Inc., 2020).

[5] Gao, Y. et al. *Retrieval-Augmented Generation for Large Language Models: A Survey*. 10.48550/arXiv.2312.10997 (2023).

[6] Du, Y. et al. Improving biomedical question answering by data augmentation and model weighting. *Ieee-Acm Trans. Comput. Biology Bioinf.* **20**(2), 1114–1124 (2023). 10.1109/TCBB.2022.3171388 35486563

[7] Yalamanchili, A. et al. Quality of large Language model responses to radiation oncology patient care questions. *JAMA Netw. Open.*, **7**(4) (2024). 10.1001/jamanetworkopen.2024.4630 PMC10988356 38564215

[8] Caruccio, L. et al. Can ChatGPT provide intelligent diagnoses? A comparative study between predictive models and ChatGPT to define a new medical diagnostic bot. *Expert Syst. Appl.* **2352024).**

[9] Hu, D. et al. Zero-shot information extraction from radiological reports using ChatGPT. *Int. J. Med. Inform.* **183** (2024). 10.1016/j.ijmedinf.2023.105321 38157785

[10] Fonseca, A. et al. Embracing the future-is artificial intelligence already better? A comparative study of artificial intelligence performance in diagnostic accuracy and decision-making. *Eur. J. Neurol.* **31**(4) (2024). 10.1111/ene.16195 PMC11235701 38235841

[11] Wang, L. et al. An entity extraction pipeline for medical text records using large language models: analytical study. *J. Med. Internet. Res.* **26**, e54580–e54580 (2024). 38551633 10.2196/54580 PMC11015372

[12] Ying, L. et al. Screening/diagnosis of pediatric endocrine disorders through the artificial intelligence model in different language settings. *Eur. J. Pediatr.* (2024). 10.1007/s00431-024-05527-1 PMC11098926 38502320

[13] Walker, H. L. et al. Reliability of medical information provided by chatgpt: assessment against clinical guidelines and patient information quality instrument. *J. Med. Internet. Res.* **252023).** 10.2196/47479 PMC10365578 37389908

[14] Bernstein, I. A. et al. Comparison of ophthalmologist and large Language model chatbot responses to online patient eye care questions. *Jama Netw. Open.* **6**(8) (2023). 10.1001/jamanetworkopen.2023.30320 PMC10445188 37606922

[15] Yang, J. et al. Chat generative pretrained transformer (ChatGPT) and bard: artificial intelligence does not yet provide clinically supported answers for hip and knee osteoarthritis. *J. Arthroplast.* **39**(5), 1184–1190 (2024). 10.1016/j.arth.2024.01.029 38237878

[16] Nastasi, A. J. et al. A vignette-based evaluation of ChatGPT’s ability to provide appropriate and equitable medical advice across care contexts. *Sci. Rep.* **13**(1) (2023). 10.1038/s41598-023-45223-y PMC10587094 37857839

[17] Gracias, D. et al. Exploring the role of an artificial intelligence chatbot on appendicitis management: an experimental study on ChatGPT. *ANZ J. Surg.* **94**(3), 342–352 (2024). 37855397 10.1111/ans.18736

[18] Eckrich, J. et al. Urology consultants versus large language models: potentials and hazards for medical advice in urology. *Bjui Compass*. (2024). 10.1002/bco2.359 PMC11090772 38751951

[19] Wu, C. et al. PMC-LLaMA: Toward Building open-source language models for medicine. *J. Am. Med. Inform. Assoc.* (2024). 10.1093/jamia/ocae045 PMC11639126 38613821

[20] Xue, X. et al. Xiaoqing: A Q&A model for glaucoma based on LLMs. *Comput. Biol. Med.* **174**, 108399–108399 (2024). 38615461 10.1016/j.compbiomed.2024.108399

[21] Miao, J. et al. Integrating retrieval-augmented generation with large language models in nephrology: advancing practical applications. *Medicina-Lithuania*. **60**(3) (2024). 10.3390/medicina60030445 PMC10972059 38541171

[22] Gilbert, S., Kather, J. N. & Hogan, A. Augmented non-hallucinating large language models as medical information curators. *Npj Digit. Med.* **7**(1) (2024). 10.1038/s41746-024-01081-0 PMC11039648 38654142

[23] Zakka, C. et al. Almanac: retrieval-augmented language models for clinical medicine. *Res. Sq.* (2023). 10.1056/aioa2300068 PMC10857783 38343631

[24] Kresevic, S. et al. Optimization of hepatological clinical guidelines interpretation by large Language models: a retrieval augmented generation-based framework. *Npj Digit. Med.* **7**(1) (2024). 10.1038/s41746-024-01091-y PMC11039454 38654102

[25] Safrai, M. & Azaria, A. Does small talk with a medical provider affect chatgpt’s medical counsel? Performance of ChatGPT on USMLE with and without distractions. *PLOS ONE*. **194), e0302217 (2024).** 38687696 10.1371/journal.pone.0302217 PMC11060598

[26] Kruse, C. S. et al. Challenges and opportunities of big data in health care: A systematic review. *JMIR Med. Inf.* **44), e38 (2016).** 10.2196/medinform.5359 PMC5138448 27872036

[27] Lin, C. Y. Rouge: A package for automatic evaluation of summaries. In *Text Summarization Branches Out*. (2004).

[28] Papineni, K. et al. Bleu: a method for automatic evaluation of machine translation. In *Proceedings of the 40th annual meeting of the Association for Computational Linguistics*. (2002).

[29] Zhang, T. et al. *BERTScore: Evaluating Text Generation with BERT.* ArXiv, abs/1904.09675. (2019).

[30] Hurley, E. T. et al. Evaluation High-Quality of information from ChatGPT (Artificial intelligencedlarge Language Model) artificial intelligence on shoulder stabilization surgery. *Arthrosc. J. Arthroscopic Relat. Surg.*, **40**(3). (2024). 10.1016/j.arthro.2023.07.048 37567487

